# RETRACTION: Probucol Protects Rats from Cardiac Dysfunction Induced by Oxidative Stress following Cardiopulmonary Resuscitation

**DOI:** 10.1155/omcl/9812128

**Published:** 2026-04-08

**Authors:** Oxidative Medicine and Cellular Longevity

RETRACTION: X. Xiao, H. Hou, V. Lin, et al., “Probucol Protects Rats from Cardiac Dysfunction Induced by Oxidative Stress following Cardiopulmonary Resuscitation,” *Oxidative Medicine and Cellular Longevity*, 2017, 1284804, https://doi.org/10.1155/2017/1284804.

The above article, published online on 30 October 2017 in Wiley Online Library (wileyonlinelibrary.com), has been retracted by agreement between the Journal Editor‐in‐Chief, Jeannette Vasquez‐Vivar; and John Wiley & Sons Ltd. The retraction has been agreed due to raised concerns with the figures. Specifically:

• In Figure 3, all panels are highly similar to five of the panels presented in Figure 5a of [1]
• In Figure 4, all panels are highly similar to five of the panels presented in Figure 6a of [1]

After assessment of the concerns and the author’s response, the editorial board have determined that the results and conclusions can no longer be considered reliable, and the article is therefore retracted.

Adam May agrees to the retraction. No response was received from the remaining authors.

## References

[1] Xu Q. , Zhou Y. , Zhang R. , Sun Z. , and Cheng L.-F. , Antiarthritic Activity of Qi-Wu Rheumatism Granule (a Chinese Herbal Compound) on Complete Freund’s Adjuvant-Induced Arthritis in Rats, Evidence-Based Complementary and Alternative Medicine. (2017) 2017, no. 1, 10.1155/2017/1960517, 2-s2.0-85042216653, 1960517. 29238384 PMC5697382

